# Supplementary material for: Genomic Analysis Reveals New Integrative Conjugal Elements and Transposons in GBS Conferring Antimicrobial Resistance
Source: Antibiotics (Basel). 2023 Mar 9;12(3):544. doi: 10.3390/antibiotics12030544 (PMC10044541; doi:10.3390/antibiotics12030544)
Supplement: Supplementary file 1 [file antibiotics-12-00544-s001.zip › antibiotics-2228124-supplementary.pdf]

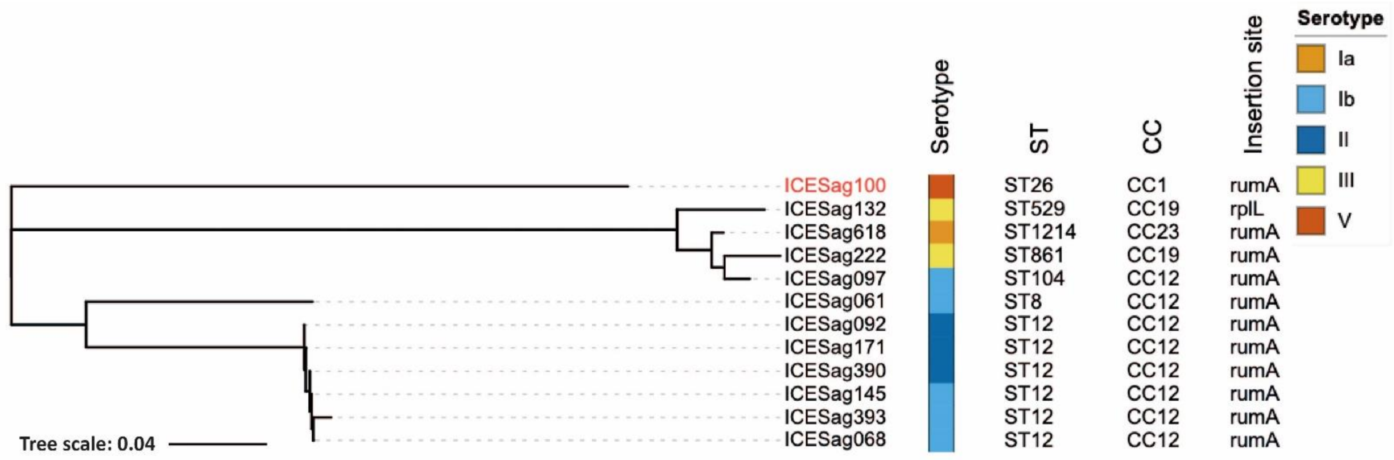

Figure S1. Neighbour joining phylogeny for the *tet*O alone carrying ICEs. Ten *tet*O ICEs were located at the *rum*A, and for one isolate the insertion site of ICE was *rpl*L. The ICESag100 (accession no. OP715836) carrying *tet*W shown in red was included as an outgroup and was also inserted at *rum*A. Information of serotype, sequence type (ST), Clonal complex (CC) and insertion site of ICE are also provided next to the phylogeny.

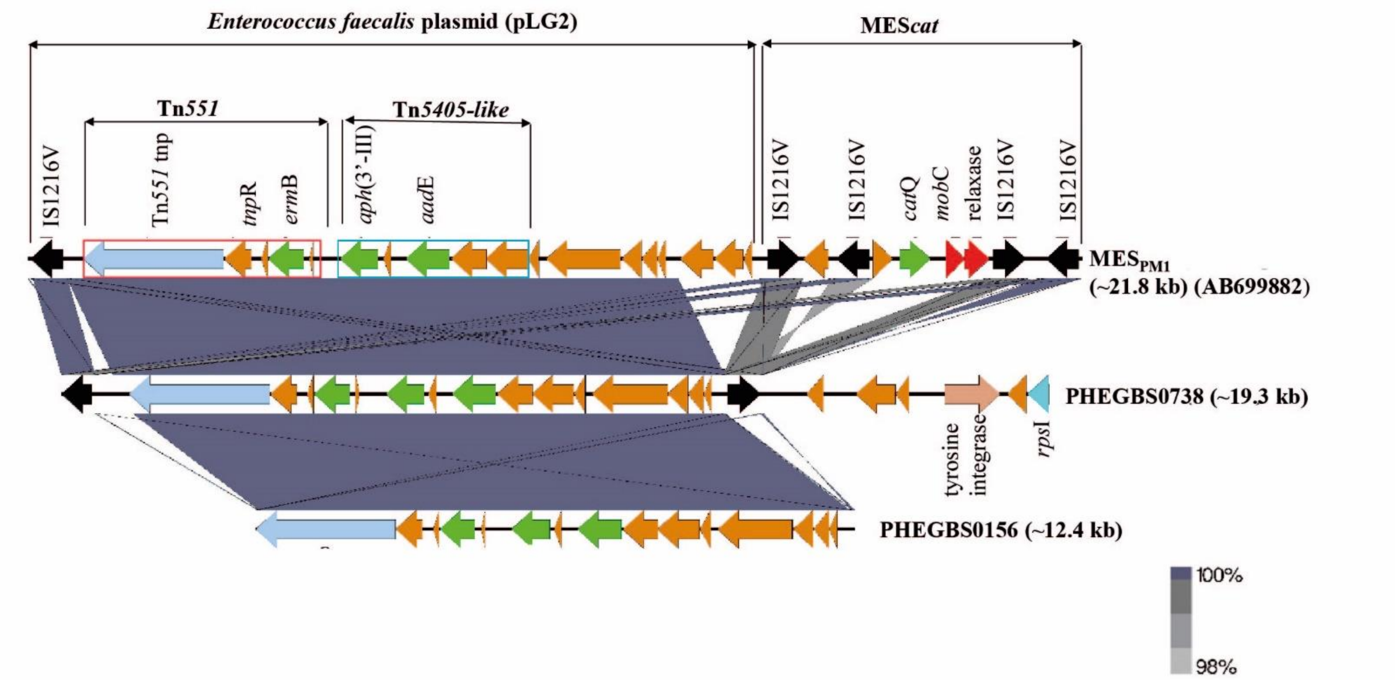

Figure S2. Comparison of *Staphylococcus aureus* composite mobile element structure MES$_{PM1}$ to CC19 GBS isolates. Two isolates PHEGBS0738 – V/ST19 and PHEGBS0156 – III/ST19 found to carry ARGs by MES$_{PM1}$ (AB699882) and reduced MES$_{PM1}$, respectively. Blastn was performed using Easyfig v2.2.2. Different colours are indicating important features where light blue showing Tn551 transposon (tnp) gene, black showing insertion sequences - IS1216V, green - antibiotic resistance genes, red - conjugal proteins. pink-tyrosine integrase and orange other significant genes of MES$_{PM1}$. Areas with BLAST hits shown by bars between sequences in grey colour. Genomic size of MES$_{PM1}$ is ~21.8 kb.

Table S1. Substitutions in five Penicillin binding proteins (PBPs) identified in 193 GBS isolates relative to the reference PBPs. The reference PBPs of *S. agalactiae* 2603V/R (NC_004116) were used for this analysis. “NIL” represents no substitution observed and “-” represents gap observed.

**Penicillin binding protein types**	**Reference protein id for PBPs**	**Similarity (%) to reference PBPs**	**No. of isolates**	**Substitutions** (none confer penicillin resistance and likely represent non-resistance polymorphisms)
**PBP1a**	NP_687333.1	100	6	NIL
		99.98	1	S685N, N702D, G739_ G741del, V746A
		98.93	2	A27T, A734V, G739_N743del, V746A
		98.8	1	ins54S55S, S473N, N702D, N740_N743del, V746A
		99.73	77	G739_N740del
		99.6	6	R662H, G739_N740del
		99.47	14	N740_G743del
			1	G739_ N742del
		99.33	2	N740_ G743del, F544V
		99.2	3	S685N, G739_ N742del, V746A
		99.07	76	N740_ N743del, V746A
			1	G739_ N742del
		99.02	3	A27T, G739S, N740_ N743del, V746A
		**Total**	**193**	
**PBP1b**	NP_687194.1	100	62	NIL
		99.87	59	A36D (n=48); L41S (n=12)
		99.74	66	A95D + D505E (n=4); A36D + L46S (n=59); A95D + L41S (n=3)
		99.73	3	A36D, I46S
		99.52%	3	A95D
		**Total**	**193**	
**PBP2a**	NP_689052.1	100	80	NIL
		99.96	1	A581T
		99.74	43	E63K, N666K, V761L
		99.61	27	P69L, K316Q, R345Q
		99.57	37	E63K
		99.53	1	E63K, V549
		99.48	2	P69L, D313N, K316Q, R345Q
		99.40%	1	P69L, K316Q, R345K
		99.35%	1	D18N, L317V
		**Total**	**193**	
**PBP2b**	NP_687780.1	100	8	NIL
		99.87	168	1Mdel, L2M

		99.8	13	1Mdel, L2M, V80A
		99.76	3	V80A
		99.6	1	A69V, V80A, G470S
		**Total**	**193**	
**PBP2x**	NP_687322.1	100	8	NIL
		99.78	88	I377V
		99.2	2	I377V, V510I
		99.11%	1	I377V, V510I, K570E
		95.21	11	1M_A35del, I377V
		95.08	79	1M_A35del, I377V, V510I
		94.95	4	1M_A35del, Y366H, I377V
		**Total**	**193**	
